# Even Mild Hyperlactatemia Is Associated with Increased Mortality in Critically Ill Patients

**DOI:** 10.1186/cc12891

**Published:** 2013-09-11

**Authors:** Asgar H Rishu, Raymond Khan, Hasan M Al-Dorzi, Hani M Tamim, Saad Al-Qahtani, Ghassan Al-Ghamdi, Yaseen M Arabi

**Affiliations:** 1 Intensive Care Department, King Saud bin Abdulaziz University for Health Sciences, PO box 22490, Riyadh, Saudi Arabia, 11426; 2 King Abdullah International Medical Research Centre, King Saud bin Abdulaziz University for Health Sciences, PO box 22490, Riyadh, Saudi Arabia, 11426

## Abstract

**Introduction:**

The clinical significance of elevation of lactate levels within the reference range is not well studied. The objective of this study was to determine the best cutoff threshold for serum lactate within the reference range (0.01 to 2.00 m*M*) that best discriminated between survivors and nonsurvivors of critical illness and to examine the association between relative hyperlactatemia (lactate above the identified threshold) and mortality.

**Methods:**

This was a retrospective cohort study of adult patients admitted to the medical-surgical intensive care unit (ICU) of a tertiary care academic center. Youden index was calculated to identify the best lactate cutoff threshold that discriminated between survivors and nonsurvivors. Patients with lactate above the identified threshold were defined as having relative hyperlactatemia. Multivariate logistic regression, adjusting for baseline variables, was performed to determine the relationship between the above two ranges of lactate levels and mortality. In addition, a test of interaction was performed to assess the effect of selected subgroups on the association between relative hyperlactatemia and hospital mortality.

**Results:**

During the study period, 2,157 patients were included in the study with mean lactate of 1.3 ± 0.4 m*M*, age of 55.1 ± 20.3 years, and acute physiology and chronic health evaluation (APACHE) II score of 22.1 ± 8.2. Vasopressors were required in 42.4%. Lactate of 1.35 m*M *was found to be the best cutoff threshold for the whole cohort. Relative hyperlactatemia was associated with increased hospital mortality (adjusted odds ratio (aOR), 1.60, 95% confidence interval (CI) 1.29 to 1.98), and ICU mortality (aOR, 1.66; 95% CI, 1.26 to 2.17) compared with a lactate level of 0.01 to 1.35 m*M*. This association was consistent among all examined subgroups.

**Conclusions:**

Relative hyperlactatemia (lactate of 1.36 to 2.00 m*M*) within the first 24 hours of ICU admission is an independent predictor of hospital and ICU mortality in critically ill patients.

## Introduction

Hyperlactatemia (>2 m*M*) has been shown to be an independent predictor of mortality in different groups of critically ill patients [1,2], such as those with sepsis, with or without organ failure [3-11], trauma [12-17], and acute inflammatory response syndrome [7,18]. It has been also used to guide management of critically ill patients. For example, the Surviving Sepsis Campaign [19] and early goal-directed therapy [20] recommend resuscitation of sepsis patients with lactate >4 m*M*. In clinical practice, less attention is paid to lactate levels within the reference range (≤2 m*M*), as their clinical significance is not well understood. Studies have suggested that relative hyperlactatemia in patients with septic shock has been associated with mortality [21-23], indicating that lactate might remain normal before and during resuscitation, thus questioning what is the best cutoff threshold of lactate that should trigger resuscitation. However, data in this area remain limited. Therefore, we sought to determine the best cutoff threshold for serum lactate that is within the reference range (0.01 to 2.00 m*M*) that best discriminates between survivors and nonsurvivors of critical illness and to examine the association between relative hyperlactatemia (lactate above the identified threshold) and mortality.

## Methods

### Design and Setting

This was a retrospective cohort study of adult patients admitted to the medical-surgical intensive care unit (ICU) of King Abdulaziz Medical City, a tertiary care academic center in Riyadh, Saudi Arabia, from February 2002 until December 2010. The hospital is accredited by the Joint Commission International. The ICU is a 21-bed closed unit with a 24 hours/7 days in-house coverage by board-certified intensivists [24] and admits around 900 patients per year. The ICU has a database for all patients for which data was prospectively collected by a full-time research physician. The study was approved by the institutional review board of the hospital and, because of the nature of the study, consent was waived.

### Patients

All patients 18 years of age or older with measured lactate levels were included in this study. We excluded patients with readmission within the same hospitalization and brain death on admission. The following information were noted: age, gender, acute physiology and chronic health evaluation (APACHE) II score [25], admission diagnosis category as defined by the acute physiology and chronic health evaluation (APACHE) II system [25], chronic comorbidities, history of cirrhosis, history of diabetes, Glasgow coma scale (GCS), presence of sepsis on admission, mechanical ventilation requirement in the first 24 hours of admission, the ratio of partial pressure of oxygen to the fraction of inspired oxygen (PaO_2_/FiO_2_), requirement for vasopressors (defined as use of any vasopressor infusion except dopamine <5 μg/kg/min), admission bilirubin, creatinine, lactate, and international normalized ratio (INR), ICU and hospital length of stay (LOS), mechanical ventilation duration (MVD), requirement for tracheostomy and renal-replacement therapy (RRT), and ICU and hospital mortality.

### Outcomes

Hospital mortality was the primary outcome. ICU mortality, ICU and hospital LOS, MVD and requirement for tracheostomy, and RRT were the secondary outcomes.

### Measurement of lactate

Lactate was measured on admission to ICU by sending venous or arterial blood to the hospital central laboratory and was measured via Abbott Laboratories, Abbott Park, IL 60064, USA, with a limit of detection of 0.006 m*M*. All patients who had at least one measurement of lactate within 24 hours of admission to ICU were included in the analysis. For patients with multiple lactate measurements, the highest value of lactate level within the first 24 hours of ICU admission was recorded and entered into the database, and this value was used for analysis.

### Statistical analysis

Statistical analysis software (SAS, version 9.0; SAS Institute, Cary, NC, USA) was used to analyze the data. Continuous data are presented as means with standard deviations, and categoric data, as frequencies and percentages. The χ^2 ^or Student *t *test was used to test significant differences between study groups, as appropriate. Youden index was calculated to identify the best cutoff threshold of lactate that discriminates between survivors and nonsurvivors [26]. Patients having serum lactate above the identified cutoff threshold were defined as having relative hyperlactatemia and were compared with the control group of patients with lactate below that cutoff threshold. We also calculated the Youden index among patients with sepsis and patients in shock (defined as requiring vasopressors) to determine the best cutoff in these specific groups.

To determine the association between relative hyperlactatemia and hospital mortality, bivariate and then multivariate logistic regression analyses were performed. The variables entered in the model included age, gender, APACHE II score, admission diagnosis category, history of cirrhosis, presence of sepsis on admission, mechanical ventilation, vasopressor use, INR, and RRT. The variables were selected based on statistical as well as on clinical rationales. We tested for interaction to determine the effect modification of selected subgroups on the association between relative hyperlactatemia and hospital mortality. These subgroups included the following: male versus female patients; age older than 50 years versus age younger than 50 years; sepsis versus no sepsis; diabetes versus no diabetes; different admission diagnosis categories; cirrhosis versus no cirrhosis; mechanically ventilated versus not mechanically ventilated; required vasopressors versus no vasopressors; PaFiO_2 _ratio >200 versus PaFiO_2 _ratio <200. Results were reported as adjusted odds ratio (aOR) and 95% confidence interval (CI). A two-sided *P *value <0.05 was considered to be statistically significant.

## Results

### Baseline characteristics of patients with lactate within reference range

Of 10,791 patients admitted to the ICU during the study period, 4,538 (42.1%) had lactate levels measured within 24 hours of admission to ICU. Of those with measured lactate, 2,157 (47.5%) patients had lactate levels within the reference range (0.01-2.00 m*M*). Table 1 describes the baseline characteristics of these patients. The mean age was 55.1 ± 20.3 years: 37.6% of them were female patients; APACHE II score was 22.1 ± 8.2; 22.5% had sepsis diagnosis on admission; 75% were mechanically ventilated; and 42.4% were receiving vasopressors. The mean lactate was 1.3 ± 0.4 m*M*. The overall ICU and hospital mortality were 14.1% and 30.0%, respectively.

**Table 1:** Baseline characteristics of all patients with lactate ≤ 2.00 mM

Variable	*N *= 2,157
Age (years), mean ± SD	55.1 ± 20.3
Gender, female, number (%)	810 (37.6)
APACHE II score, mean ± SD	22.1 ± 8.2
Admitting diagnostic category, number (%)	
Respiratory	537 (24.9)
Cardiovascular	579 (26.8)
Neurologic	202 (9.4)
Other medical illness	107 (5)
Nonoperative trauma	244 (11.3)
Postoperative	488 (22.6)
Chronic comorbidities, number (%)	
Chronic liver disease	195 (9.2)
Chronic cardiovascular disease	438 (20.6)
Chronic respiratory disease	389 (18.3)
Chronic renal disease	331 (15.6)
Chronic immunocompromised	228 (10.7)
Cirrhosis, number (%)	160 (7.4)
Diabetes, number (%)	914 (42.4)
GCS, mean ± SD	10.4 ± 4.3
Sepsis, number (%)	485 (22.5)
Mechanical ventilation, number (%)	1,618 (75.0)
PaO_2_/FiO_2 _ratio, number (%)	
<200	888/2,144 (41.4)
>200	1,256/2,144 (58.6)
Vasopressors, number (%)	915 (42.4)
Lab findings, mean ± SD	
Bilirubin (μ*M*)*	41.0 ± 98.6
Creatinine (μ*M*)*	158.2 ± 162.2
Lactate (m*M*)*	1.3 ± 0.4
INR	1.4 ± 0.7
ICU LOS (days), mean ± SD	9.2 + 10.8
Hospital LOS (days), mean ± SD	52.0 ± 75.8
Mechanical ventilation duration (days), mean ± SD	7.5 ± 10.3
Tracheostomy, number (%)	403 (18.7)
RRT, number (%)	366 (17.0)
ICU mortality, number (%)	304 (14.1)
Hospital mortality, number (%)	648 (30.0)

APACHE II: acute physiology and chronic health evaluation; PaO_2_/FiO_2_, the ratio of partial pressure of oxygen to the fraction of inspired oxygen; GCS, Glasgow coma scale; INR, International normalized ratio; ICU, intensive care unit; LOS, length of stay; RRT, renal replacement therapy. To convert bilirubin to mg/dl, divide by 17.1; creatinine to μg/dl, divide by 88.4; lactate to g/dl, divide by 0.111

### Youden index

Table 2 summarizes the sensitivities and specificities, along with the 95% CI, for different lactate cutoff points among patients with lactate within the reference range. Moreover, Youden index for each of these cutoff points is presented. The best cutoff threshold that was found to discriminate between survivors and nonsurvivors was 1.35 m*M*, as it corresponded to the highest Youden index.

**Table 2:** Youden index calculation in the whole cohort of patients with lactate within the reference range

Lactate level (m*M*)	Sensitivity	95% CI	Specificity	95% CI	LR+	LR-	Youden index
≥0.07	100.00	99.4-100.0	0.00	0.0-0.2	1.00		0
>0.09	100.00	99.4-100.0	0.13	0.02-0.5	1.00	0.00	0.0013
>0.1	99.85	99.1-100.0	0.20	0.04-0.6	1.00	0.78	0.0005
>0.2	99.54	98.7-99.9	0.40	0.1-0.9	1.00	1.16	-0.0006
>0.3	99.07	98.0-99.7	0.53	0.2-1.0	1.00	1.75	-0.004
>0.4	98.61	97.4-99.4	1.06	0.6-1.7	1.00	1.31	-0.0033
>0.5	97.69	96.2-98.7	2.78	2.0-3.7	1.00	0.83	0.0047
>0.6	95.52	93.6-97.0	6.76	5.5-8.1	1.02	0.66	0.0228
>0.7	92.90	90.6-94.8	12.39	10.8-14.2	1.06	0.57	0.0529
>0.8	85.96	83.0-88.5	18.82	16.9-20.9	1.06	0.75	0.0478
>0.9	79.17	75.8-82.2	25.78	23.6-28.1	1.07	0.81	0.0495
>1	70.52	66.8-74.0	36.71	34.3-39.2	1.11	0.80	0.0723
>1.1	62.35	58.5-66.1	45.79	43.3-48.3	1.15	0.82	0.0814
>1.2	57.41	53.5-61.3	54.14	51.6-56.7	1.25	0.79	0.1155
>1.3	51.08	47.2-55.0	62.16	59.7-64.6	1.35	0.79	0.1324
>1.4	41.51	37.7-45.4	69.18	66.8-71.5	1.35	0.85	0.1069
>1.5	32.87	29.3-36.6	74.82	72.5-77.0	1.31	0.90	0.0769
>1.6	25.77	22.4-29.3	80.91	78.8-82.9	1.35	0.92	0.0668
>1.7	19.44	16.5-22.7	85.22	83.3-87.0	1.32	0.95	0.0466
>1.8	11.73	9.4-14.5	89.86	88.2-91.3	1.16	0.98	0.0159
>1.9	6.02	4.3-8.1	94.76	93.5-95.8	1.15	0.99	0.0078
>2	0.00	0.0-0.6	100.00	99.8-100.0		1.00	0

CI, confidence interval.

LR+, Likelihood positive ratio = sensitivity/(1-specificity).

LR-, Likelihood negative ratio = (1-sensitivity)/specificity).

### Baseline characteristics of patients with relative hyperlactatemia

Compared with control group, patients with relative hyperlactatemia were older, had higher APACHE II scores, were more likely to have chronic liver disease and sepsis on admission, were more likely to be mechanically ventilated and to be receiving vasopressors, and had higher INRs (Table 3).

**Table 3:** Baseline characteristics of patients with relative hyperlactatemia (lactate, 1.36 to 2.00 m*M*) compared with the control group (lactate, 0.01 to 1.35 m*M*)

	Control group *n *= 1,255	Relative hyperlactatemia *n *= 902	*P *value
Age (years), mean ± SD	54.1 ± 20.7	56.5 ± 19.7	0.009
Gender, female, number (%)	478 (38.1)	332 (36.8)	0.54
APACHE II, mean ± SD	21.5 ± 8.0	22.8 ± 8.4	0.0004
Admitting diagnostic category, number (%)			
Respiratory	324 (25.8)	213 (23.6)	0.04
Cardiovascular	312 (24.8)	267 (29.6)	
Neurologic	123 (9.8)	79 (8.8)	
Other medical illness	67 (5.3)	40 (4.4)	
Nonoperative trauma	157 (12.5)	87 (9.7)	
Postoperative	272 (21.7)	216 (24.0)	
Chronic comorbidities, number. (%)			
Chronic liver disease	98 (8.0)	97 (10.7)	0.02
Chronic cardiovascular disease	250 (20.8)	188 (21.1)	0.61
Chronic respiratory disease	220 (17.8)	169 (19.0)	0.51
Chronic renal disease	194 (15.7)	137 (15.4)	0.81
Chronic immunocompromised	133 (10.8)	95 (10.7)	0.93
Cirrhosis, number (%)	82 (6.5)	78 (8.7)	0.06
Diabetes, number (%)	530 (42.2)	384 (42.6)	0.87
GCS, mean ± SD	10.6 ± 4.2	10.2 ± 4.4	0.07
Sepsis, number (%)	256 (20.4)	229 (25.4)	0.006
Mechanical ventilation, number (%)	917 (73.1)	701 (77.7)	0.01
PaO_2_/FiO_2 _ratio, number (%)			
<200 mm Hg	499/1247 (40.0)	389/897 (43.4)	0.12
>200 mm Hg	748/1247 (60.0)	508/897 (56.6)	
Vasopressors, number (%)	508 (40.5)	407 (45.1)	0.03
Laboratory findings, mean ± SD			
Bilirubin, (μ*M*)	38.2 ± 101.4	44.9 ± 94.6	0.13
Creatinine, (μ*M*	157.8 ± 169.3	158.8 ± 151.9	0.88
Lactate (m*M*)	1.0 ± 0.2	1.7 ± 0.2	<0.0001
INR	1.3 ± 0.7	1.4 ± 0.7	0.01

APACHE, acute physiology and chronic health evaluation; PaO_2_/FiO_2, _the ratio of partial pressure of oxygen to the fraction of inspired oxygen; GCS, Glasgow Coma Scale; INR, International normalized ratio. To convert bilirubin to mg/dl divide by 17.1; creatinine to mg/dl by 88.4; lactate to mg/dl, divide by 0.111.

### Outcomes of patients with relative hyperlactatemia

Hospital mortality was higher in patients with relative hyperlactatemia compared with the control group (36.7% versus 25.3%; *P *< 0.0001). Similarly, ICU mortality was higher (18.4% versus 11.0%; *P *= 0.0003) (Table 4). On multivariate logistic regression analysis, relative hyperlactatemia was an independent predictor of hospital mortality (aOR, 1.60; 95% CI, 1.29 to 1.98), and ICU mortality (aOR, 1.66; 95% CI, 1.26 to 2.17) (Table 4).

**Table 4:** Outcomes in patients with lactate levels within reference range

Variable	Control group *n *= 1,255	Relative hyperlactatemia *n *= 902	aOR (95% CI)	*P *value
Hospital mortality, number (%)	317 (25.3)	331 (36.7)	1.60 (1.29-1.98)	<0.0001*
ICU mortality, number (%)	138 (11.0)	166 (18.4)	1.66 (1.26-2.17)	0.0003*
ICU LOS, mean ± SD	9.1 ± 10.4	9.4 ± 11.4	*	0.49
Hospital LOS, mean ± SD	52.6 ± 71.6	51.2 ± 81.4	*	0.69
Mechanical ventilation duration mean ± SD	7.3 ± 10.3	7.8 ± 10.3	*	0.32
Tracheostomy, number. (%)	246 (19.6)	157 (17.4)	0.78 (0.62-0.98)	0.04*
RRT, number (%)	202 (16.1)	164 (18.2)	1.16 (0.92-1.45)	0.20*

ICU, Intensive care unit; LOS, length of stay; RRT, renal replacement therapy.

*Adjusted for age, gender, APACHE II score, admission diagnosis category, mechanical ventilation, cirrhosis, presence of sepsis on admission, vasopressor use, INR, and RRT.

### Subgroup analysis

**Figure 1:**
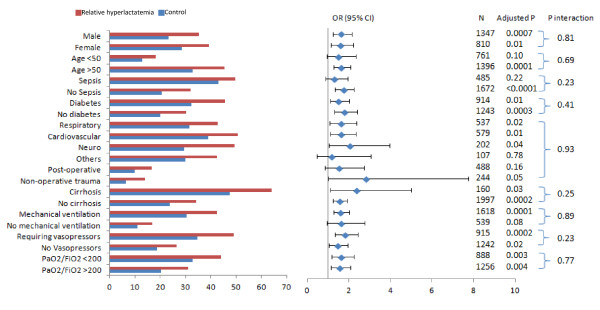
**Crude mortality, adjusted odds ratios, confidence intervals, and *P *value for the test of interaction for the association between relative hyperlactatemia and hospital mortality in different subgroups**.

The association between relative hyperlactatemia on hospital mortality in different subgroups is shown in Figure 1. Relative hyperlactatemia was associated with increased hospital mortality, which was consistent among all subgroups, as indicated by the test of interaction. Youden index calculations showed that the cutoff threshold among patients with sepsis and those who were in shock was not different from that of the whole population.

## Discussion

Among critically ill patients with lactate levels within the reference range (0.01 to 2.00 m*M*), we found lactate of 1.35 m*M *to be the best cutoff threshold that discriminated between survivors and nonsurvivors. Relative hyperlactatemia was an independent predictor of hospital and ICU mortality.

The first demonstration of lactate in human blood was in shock patients, by Johann Joseph Scherer in 1843 [27]. Subsequent work led to the demonstration that tissue hypoxia was associated with increased lactate level [27]. Elevated lactate is frequently seen in critically ill patients and is primarily a consequence of inadequate oxygen delivery, usually associated with significant cardiopulmonary compromise, as seen in cardiogenic, hypovolemic, and septic shock [28].

In an unstressed individual, the basal lactate concentration is 1.0 ± 0.5 m*M*. In critically ill patients, a lactate level of ≤2 m*M *is considered to be within the reference range [29], and therefore, hyperlactatemia is defined as lactate of >2 m*M*. A wealth of literature exists on hyperlactatemia (>2 m*M*) [2,4,6,10] but not on lactate within the reference range.

We found that a significant percentage (47.5%) of critically ill patients who had lactate measured within 24 hours of ICU admission had an admitting lactate level of ≤2 m*M*, with overall hospital mortality of 30%. It has been reported that 24% to 34% of patients with severe sepsis and septic shock have lactate levels within the reference range [10,22]. Trzeciak *et al*. [30] evaluated 1,177 patients with primary or secondary diagnosis of infection and had serum lactate measured and found that most (70.3%) patients had lactate of 0.0 to 2.0 m*M *[30].

The evidence linking lactate within the reference range (0.01 to 2.0 m*M*) to mortality is also limited. We found that relative hyperlactatemia was an independent predictor of both hospital and ICU mortality. Nichol *et al*. [31], in their prospective observational study of 7,155 consecutive critically ill patients, found that increased admission lactate within the reference range was independently associated with increased hospital mortality in general critically ill patients. Trzeciak *et al*. [30] found that patients with infections and lactate <2 m*M *had a mortality of 15% compared with 25% for patients with lactate 2.1 to 3.9 m*M *and 38% for those with lactate ≥4 mM (*P *< 0.001). In our study, we found that a lactate of 1.35 m*M *was the best cutoff threshold to discriminate between survivors and nonsurvivors among critically ill patients who had lactate within the reference range. Interestingly, this cutoff threshold was similar in patients with sepsis and also in patients with shock. This may be of significant importance in identifying severe sepsis early, which is one of the main targets of sepsis-management quality-improvement initiatives.

Whether patients with relative hyperlactatemia should be treated differently is unknown. Rivers *et al*. [20], in the early goal-directed therapy study for severe sepsis or septic shock, excluded patients with MAP >65 mm Hg and lactate <4 m*M *from early intervention. The Surviving Sepsis Campaign Guidelines also recommended lactate of >4 m*M *(even in the absence of hypotension) as one of the criteria for initiating early-goal-directed therapy [19]. Whether early intervention in patients with relative hyperlactatemia will improve outcomes is a question that may be intuitive but requires further study.

Our study should be viewed in terms of its strengths and limitations. Strengths include the analysis of prospectively collected data by a full-time research physician, large sample size, 24/7 coverage of the unit by board-certified intensivists [24] rendering uniform care to all patients. The lactate was measured in the central laboratory, thus minimizing possible false readings of various point-of-care devices because of different methods of measurements used in many ICUs [32]. As for limitations, the study is single centered, lactate levels were not measured in all patients, and the analysis was performed by using a single, not serial values. Finally, we do not have data about the interventions and medications that can affect the lactate level.

## Conclusions

Relative hyperlactatemia (lactate of 1.36 to 2.00 m*M*) within the first 24 hours of ICU admission is an independent predictor of hospital and ICU mortality in critically ill patients. Further studies are required to examine the most appropriate diagnostic and therapeutic approach to patients with relative hyperlactatemia.

## Key messages

• Among critically ill patients with lactate within the reference range, lactate of 1.35 m*M *was found to be the best cutoff threshold that discriminated between survivors and nonsurvivors.

• Relative hyperlactatemia within the first 24 hours of ICU admission is an independent predictor of hospital and ICU mortality in critically ill patients.

• Association between hospital mortality and relative hyperlactatemia was consistent among all subgroups, including patients with sepsis and those with shock.

## Abbreviations

aOR: adjusted odds ratio; APACHE: acute physiology and chronic health evaluation; CI: confidence interval; GCS: Glasgow Coma Score; ICU: intensive care unit; INR: International normalized ratio; LOS: length of stay; MVD: mechanical ventilation duration; PaO_2_/FiO_2_: ratio of partial pressure of oxygen to the fraction of inspired oxygen; RRT: renal replacement therapy; SAS: statistical analysis software.

## Competing interests

The authors declare that they have no competing interests.

## Authors' contributions

AHR participated in conception and design, participated in analysis and interpretation of data, drafted the manuscript, critically revised the manuscript for important intellectual content, and approved the final version to be published. RK participated in analysis and interpretation of data, helped to draft the manuscript, critically revised the manuscript for important intellectual content, and approved the final version to be published. HMA participated in analysis and interpretation of data, critically revised the manuscript for important intellectual content, and approved the final version to be published. HMT helped in statistical analysis and interpretation of data, critically revised the manuscript for important intellectual content, and approved the final version to be published. SAQ participated in conception and design, helped in drafting the manuscript, critically revised the manuscript for important intellectual content, and approved the final version to be published. GAG participated in conception and design, critically revised the manuscript for important intellectual content, and approved the final version to be published. YMA participated in conception and design, acquisition of data, analysis and interpretation of data, critically revised the manuscript for important intellectual content, and approved the final version to be published.
